# Stricture of the Urethra, by the Electrical Treatment

**Published:** 1874-10-15

**Authors:** A. J. Steele


### Gleanings from Our Exchanges.

### STRICTURE OF THE URETHRA BY THE ELECTRICAL TREATMENT.

BY A. J. STEELE, M.D.

*From the Western Lancet, Sept.*, 1874.

THE attention of the profession has been of late especially called, and very justly, to a comparatively new method of treating strictures of the urethra, namely, by the use of galvanism. The ease of the application, the slight inconvenience to the patient, and the rapidity and permanence of the cure, make it really deserving of a prominent place among the surgical advances of the day. As my own experience corroborates the favorable reports that have been made in regard to it, I cheerfully add testimony in its favor.

The form of electricity used is that of the continuous current, and tension is sought rather than quantity, so that many small cups are demanded rather than a few large ones. I have usually found that from ten to fourteen pairs of the zinc-carbon elements have generated sufficient electricity for the purpose. The negative electrode is a metallic point pressed gently against the stricture; the positive electrode a moist sponge placed anywhere upon the surface of the body, though I have believed the action to be more energetic when it has been placed near the negative pole, as to the iliac region or thigh, rather than remotely, as to the leg or palm of the hand.

A metallic oval tip, connected to a wire passing through a gum catheter, is the form of bougie recommended, and which I have used; but I now prefer the ordinary conical steel bougie. A set, including all sizes, makes the convenience of application greater, and being silver or nickel-plated prevents oxidation. The instrument is insulated to within an inch of the point by the application of a coating of collodion; Squibbs' flexible I find well adapted for the purpose.* A *sene-fine* affords an eligible method of connecting the wire to the handle—not coated—of the bougie.

Two factors enter into the thoroughness and rapidity with which a cure can be effected, viz., the electro-motive force used, and the character of the structure to be acted upon. The softer, the more moist and vascular the stricture, the more readily will it be decomposed and absorbed; whereas extremely hard tissue will demand increased time and greater tension, and possibly, also, increased quantity. Though in regard to the latter I am prepared to believe that mistakes have been made, and failures recorded, from its injudicious use. Quantity gives a calorific effect, with rapid destruction of tissue, as in the case of the galvanic cautery; the scar resulting therefrom would be highly prejudicial in the instance of a stricture. It is rather the electrolytic action that is desirable, whereby the organic structure is disintegrated,—decomposed. The negative pole attracts hydrogen, and gives an alkaline reaction when acting upon moist animal tissues, chemically decomposing,—dissolving the part, and doubtless, too, by its stimulant action inducing absorption.

* Ether will dissolve it off when desired.

The situation and character of the stricture having been accurately determined, a bougie, prepared as above, and of a few sizes greater in calibre than the stricture, is introduced down to the obstruction, and connected by its free end to the negative wire. The sponge, moistened with salt water, placed externally on the skin—the thigh or iliac region being convenient—is attached to the positive wire. It is best to commence with a single pair, and gradually increase the number of cups, as thereby the parts are more tolerant; a low power gradually benumbing, a high power unpleasantly shocking. If the sponge is shifted without being removed from the surface, the pricking or burning sensation ordinarily experienced will be lessened. The sensations of the patient will, to some extent, determine how high a power may be used; from ten to fourteen pairs, as before remarked, may be all-sufficient, if the battery is working well. The character of the stricture, also, necessarily enters into this question. A few moments' gentle pressure and the instrument is found to pass gradually on. Once well entered, the bougie is retained *in situ*, the action being continued for a few moments longer. The current may now be gradually diminished, and the wire disconnected, the instrument retained, and, if gentle force will accomplish it, pushed on into the bladder. If not interdicted by local inflammation, the operation may be repeated in a week or fortnight's time, followed up by the careful and judicious use of bougies. In some cases one application is sufficient; in others several seances are required, depending on the character of the stricture.

Results have been most satisfactory. Strictures, accompanied with incontinence of urine, gleety discharge, irritability of bladder, painful micturition, etc., being entirely removed and rapidly cured.

Danger in this operation is reduced to a shadow, if too great quantity and too prolonged application are avoided. Care, also, in the after use of bougies is to be regarded.

While there is much of merit in the old ways, let us not be too chary in trying the new.

---

ACNE ROSACEA.—Dr. W. B. Cheadle (*Practitioner*, July) takes Hebra's view of the pathology of this affection, in opposition to that of Wilson and Tilbury Fox. He does not regard it as an acne, the sebaceous glands being neither primarily affected nor in many instances involved at all. The essential morbid change does not consist in any inflammatory process, but in a new formation of vascular and connective tissues, the changes in the sebaceous glands being secondary or accidental. The conditions associated with its production are usually excessive indulgence in alcoholic drinks, gastric disorder, uterine derangements, and prolonged or frequent exposure of the face to heat or cold.

The manner in which these causes produce the particular effects observed is, Dr. C. thinks, by long continued hyperæmia brought about by reflex action upon the local vascular system. An example of transient reflex action is seen in flushing of the face after a hearty meal or alcoholic indulgence; and it is this same effect persistently exercised which brings about morbid changes. Of course, as there is no true local inflammation, local remedies are generally useless. Stimulating applications—lotions of the perchloride of mercury, of sulphur, or of both, and applications of the acid nitrate of mercury—are the only local remedies which Dr. C. has found advantageous. Internal remedies which relieve the distention of vessels, saline purgatives for instance, are of use. Arguing from the pathology of the eruption, Dr. C. was led to apply faradization in several cases, and with the most encouraging results.

BLOOD-DRINKERS.—Upon inquiry at slaughter-houses, it is found that there are nearly two hundred persons in the city of New York who are in the habit of drinking blood flowing warmly from oxen, for strengthening purposes and for the cure of certain diseases. A lady is reported to have spoken to an inquirer as follows: "Professor Velpeau, of Paris, prescribed blood for me. I was consumptive and hastening to the grave. It has prolonged my life fifteen years. I had the utmost repugnance for it at first, but now a half-pint of hot blood from a well-conditioned ox is the greatest luxury of my life. My sister's baby so far has been preserved and nourished with little else but blood. I know twenty persons who drink it in my own neighborhood, to whom I have recommended it. It has extraordinary effects on some people, especially women, but should not be resorted to unless there is absolute weakness of the system." On a visit of the inquirer to a slaughter-house in Tenth Avenue, near Forty-second Street, he found a delicate-looking woman with a sickly boy holding a glass to the blood which ran from an ox with his throat cut. Both drank two or three glasses in turn, and departed with an appearance of added vigor. One of the butchers was asked whether he ever drank blood, and is stated to have replied to the following effect: "Sure an' I do, now; why not, now? Faith an' ye could n't tell the difference between it an' milk. It's just as swate, shure, and in the winter it's warm and foine. Bedad but it's stringthenin', sure. Hould on an' I'll get ye a drap. It's best warrum—runnin' right from the baste." The proprietor said: "All last winter we had men, women, and children every morning to drink blood. They always imbibe beast's blood; never the blood of sheep. Some of them wince a bit at first, but, when you close your eyes, blood warm from the beast's neck has just the same taste as warm milk from the cow. We do n't charge for the blood excepting when we sell it to sugar refiners." The blood of beeves is asserted to be more efficacious for weak lungs than cod-liver oil.—*Phil. Med. Times.*

INFLUENCE OF CHLOROFORM, IN LABOR, UPON THE FŒTUS. — Dr. Zweifel, of the Obstetric Clinics in Strasbourg, has recently made some investigations that would seem to imply that the anæsthetic administered to women in labor has more effect upon the fœtus in utero than is perhaps generally admitted. Dubois has made the statement that anæsthesia of the mother causes rapidity of the fœtal heart-beats. The writer has often observed an appearance of icterus upon newly born children after the use of chloroform, but could not with certainty attribute it to the latter. His attention was first seriously arrested by perceiving in the breath of an infant born a few hours before, a distinct odor of chloroform. The child had been extracted while the mother was under the influence of the anæsthetic, but since the delivery had lain in a room by itself, where no chloroform had been used. Shortly after this, in order to determine positively whether the anæsthetic was conveyed to the fœtus through the maternal circulation, he instituted the following test: a fresh placenta that had just been expelled by a woman to whom chloroform had been administered for only about fifteen minutes, and more than an hour previously, was placed in a close-fitting vessel, having first been cleansed of all adhering clots. The following day when the vessel was opened a decided odor of chloroform was perceived, and further examination proved conclusively the presence of the drug. By still another test (examination of the child's urine) the writer was able to establish the fact of the influence of the anæsthetic upon the fœtus. In conclusion, the writer observes that, since the use of narcotics in general are contraindicated in infants, it is an important question for obstetricians to decide to just what degree anæsthesia may be carried in women in labor, with impunity to the fœtus. —*Berl. Klin. Woch.*, 21, 1874.

INFLUENCE OF ALCOHOL AND TOBACCO ON THE HEART.—Nowhere can thoracic sounds be better studied than at a large recruiting depot. . . . . It is indeed curious as well as interesting to note the vagaries in cardiac sounds alone. These, although in no way so varied as the causes to which they owe their existence, are sufficiently conflicting. Thus, frequently, between exciting and depressing influences of one kind or another, it is very difficult to say how far abnormalities are ascribable to temporary and to organic derangements. Under such circumstances, cases often occur apart altogether from the morbid sounds when the heart's rhythm is perverted. I can give no better definition than a muffling of the two sounds, or what might ordinarily be called "a variety of irritable heart," occurring, however, occasionally in subjects not naturally of an excitable temperment. From these persons it was very often readily elicited that they were given to an excessive use of tobacco, either by smoking or chewing, or the two combined, accompanied, in many instances, by drunken habits. The amount of tobacco consumed daily was, ordinarily, half an ounce, and often nearly a whole ounce. From constantly observing cases of this description, and invariably associating them with the above causes, I desired several recruits to abstain entirely from tobacco and alcoholic drinks for a week, and return for inspection. In three or four instances out of ten all the symptoms disappeared; whilst in the cases where there was little or no improvement it was more than probable that the injunctions were not carried out properly. I do not know if this want of clearness in the systolic and diastolic sounds is to be detected in every instance of the excessive use of tobacco or of alcoholic drinks; but judging of the prevalence of the state in question among Londoners (chiefly in-door workmen and persons leading sedentary lives), it would seem to be pretty general.—A. Lieth Adams, M. B., F. R. S., Surgeon Major, London Recruiting District, in *The Lancet*.

EPILEPSY PRODUCED ARTIFICIALLY IN THE DOG BY SULPHATE OF QUININE.—Dr. J. Jakoubowich, of St. Petersburg *Meditzinsky Wiestnik*, 1873, Nos. 1, 2, 3, 4 and 5 (abstract in *Rev. des Sci. Medicales* by Magnan):

The injection of five to fifteen grains of sulphate of quinine in the stomach of young dogs, aged one month and a half to four months, provoked, in from forty-five to ninety minutes, apoplectic seizures. They occurred a little earlier, in from thirty to forty-five minutes, when the quinine was introduced under the skin. The attacks were variable in number, but generally in proportion to the dose. In the attack, the animal falls to the ground on its side; the pupils dilate; the mouth is fixed in trismus or remains wide open; the tongue moves to either side; the eyes turn upward; the muscles of the feet and trunk contract; the respiration is arrested. After some seconds, the clonic convulsions begin; the face becomes grimacing; the mouth opens and shuts convulsively, with gnashing of the teeth; a slimy foam shows itself on the lips; the eyelids tremble; sensibility is abolished, and the fæces escape involuntarily. After the attack follow somnolence, snoring, and often delirium; in this case the physiognomy becomes gay; the animal shows its teeth, barks, howls, wags its tail, and moves its feet, as in running. The convulsions are short, not exceeding one minute in duration.

With the epileptic attacks quinine causes vomiting, gastrorrhœa, disgust of food, feebleness of the hind limbs, and some isolated convulsions of the neck. These last accidents have been observed already for a long time, both chemically and experimentally, by many authors, who have also noticed among animals some convulsive crises, but have not expressed an opinion as to their nature. From the description given by M. Jakoubowich, these crises present the characters of epilepsy, and offer an analogy with the attacks produced by absinthe.

ELIMINATION OF ALCOHOL. — In *The Practitioner* for July, Dr. Anstie gives the results of final experiments made by himself and Dr. Dupre, with the view of ascertaining as nearly as practicable whether alcohol to any appreciable extent escapes unchanged from the body of an animal which has ingested it. The animals chosen for experiment were dogs, which approach most nearly to man in their capacity for resisting the effects of alcohol. The experiments were performed by the aid of a Pettenkofer's chamber, in which the animal was confined, while a current of air passing through the box was condensed in water. By this means *all* its excretions could be obtained and analyzed.

The result of a series of these most carefully conducted experiments, including one where the entire animal was subjected to a sort of "destructive distillation," proves conclusively that within certain limits alcohol ingested by an animal becomes totally metamorphosed within the system, the percentage eliminated as such being almost inappreciable. Dr. Anstie concludes that quite six hundred grains of absolute alcohol can be disposed of daily within the organism of an adult male without any perceptible injurious effect upon the bodily functions.

If alcohol be a force-producing food, it is probably of great value in that capacity, on account of the rapidity with which its transformations take place.

It is certain, however, that beyond a certain dosage, varying for the individual, it becomes a violent narcotic poison, the more dangerous that it cannot be eliminated to any considerable extent.

If alcohol does not disappear by oxidation, it must undergo some as yet quite unknown transformation, after which it must escape unrecognized in the excretions.

If alcohol, however, be indeed oxidized, and yet does not beget force which can be used in the system, this would be the strangest possible discovery. Considering the very high theoretical force-value of the six hundred to eight hundred grains of absolute alcohol which millions of sober persons are taking every day, we may well be hopeless of any reasonable answer to the question, Why does not this large development of wholly useless force within the body produce some violent symptoms of disturbance?

---

DIFFERENTIATION OF INTESTINAL INVAGINATION.—Dr. O. Leichtenstein, in an article on invagination (*Archiv f. Prakt. Heilk.*, 4, 1873) refers to the following points for the differentiation of invagination of the small from that of the large intestine: 1. Invagination of the small intestine but rarely occurs during the first year of life, as also rarely during childhood in general. 2. In adults, the course of the attack in invagination of the ileum is more rapid, the phenomena more severe, than in ileo-cæcal and colon invaginations. Chronic cases are rare in invaginations of the small intestine, more frequent in those of the ileo-cæcum and colon. Severe symptoms of collapse occur more frequently in the beginning of the disease. 3. Muco-sanguinolent discharges are the rule in all invaginations, whatever their seat. Fæcal evacuations, entirely normal in character (after preceding diarrhœa), were observed in ileo-cæcal invaginations, once in a colon invagination, the patient being an adult. 4. Meteorism is a very variable symptom. It is usually absent in ileo-cæcal invaginations. In invaginations of the descending colon it was frequently recognized as affecting the transverse colon, and subsequently spread over the whole abdomen. In invagination of the ileum it was occasionally found to be confined principally to the central abdominal region, with exemption of the lateral portions and epigastrium. 5. Tensemus is rare in invagination of the ileum, frequent in that of the colon and ileo-cæcum. 6. The tumor is usually absent in ileum invaginations. Its seat in the centre of the hypogastrium speaks for this variety; when situated in the cæcal region, especially when it remains stationary for some time, it indicates ileum or ileo-cæcal invagination. The spread of the tumor, when occurring suddenly and corresponding to the course of the colon, speaks more for ileo-cæcal, less for colon invagination, and excludes ileum invagination. The seat of the tumor in the left lateral portions of the abdomen would indicate ileo-cæcal or colon invagination. The tumor can never be felt in the rectum, and prolapse through the latter never occurs in uncomplicated ileum invagination. Changes in the consistency, occurrence, and disappearance of the tumor were especially observed in ileo-cæcal invagination.—*New York Medical Journal*, Sept. 1874.

---

An Unnatural Position of the Head a Cause of Death from Anæsthetics.—An interesting paper by Dr. G. W. Copeland appeared some time ago (Feb. 26, 1874) in the *Boston Medical and Surgical Journal* on the "*Styloid Muscles and Anæsthetics.*" In this article the cause of impeded breathing during anæsthesia was attributed to the action of the styloid muscles closing the glottis. It was also there shown that the difficulty could always be relieved by simply tilting the head forward so as to relax these muscles without making traction on the tonge. In a further contribution by the same writer to the *Philadelphia Medical Times* of May 30, it is claimed that death from chloroform and other anæsthetics is often caused by an unnatural position of the head, the latter being thrown back and the styloid muscles put upon the stretch. Circumstances in a number of recorded cases of death from anæsthetics are cited which favor this opinion. It is asserted " that all the deaths from nitrous oxide gas, and a large number of those from other anæsthetics, have taken place while the patients were in a sitting posture, which would allow the head to fall back farther than if they were lying down; thus favoring the theory that interference with the free action of the lungs may have been the primary cause of death." The cardiac syncope would be of course more readily induced " in patients suffering from shock or fatty degeneration, or already reduced by disease."

Another point advanced is " the importance of elevating the head sufficiently to compel the patient to inhale the anæsthetic through the nares entirely. If deep inspirations be taken through the open mouth, the lungs are inflated instantaneously, and just as rapidly emptied, leaving a long interval while no vapor is in the lungs. If the inhalations be through the nares it takes a much longer time to inflate the lungs, and a much longer time to empty them, leaving no interval. Now, the number of respirations per minute is the same either way; hence it follows that it will require a longer time to effect anæsthesia through the mouth than through the nose."—*N. Y. Medical Record.*

---

A Novel Treatment of Sciatica. — Dr. J. Prelz, Sutinsko (Bohemia), who was himself a victim of this disease, against which all therapeutic efforts have been of no avail, noticed frequently that the pain was palliated after ingestion of food, but paid no special attention to this until the fifth month of suffering. At that time the attacks were so intense and obstinate that he despaired of cure, but recollecting, casually, the observation on the effects of eating, he resolved to give this a trial. A light meal succeeded in every instance in abbreviating the attacks, but the treatment proved a very troublesome one, though successful, as many as twelve meals during the twenty-four hours being often necessary. In the sixth month the attacks ceased completely, and the patient is now well, with the exception of a chronic gastritis, the consequence of the treatment.

Dr. Prelz has since employed the same treatment successfully in two other cases.—*Wiener Med. Presse.*

